# Draft Genome Sequences of Three *Mycobacterium chimaera* Respiratory Isolates

**DOI:** 10.1128/genomeA.01409-15

**Published:** 2015-12-03

**Authors:** Micheál Mac Aogáin, Emma Roycroft, Philomena Raftery, Simone Mok, Margaret Fitzgibbon, Thomas R. Rogers

**Affiliations:** a Department of Clinical Microbiology, School of Medicine, Trinity College Dublin, St. James’s Hospital, Dublin, Ireland; b Irish Mycobacteria Reference Laboratory, LabMed Directorate, St. James’s Hospital, Dublin, Ireland

## Abstract

*Mycobacterium chimaera* is an opportunistic human pathogen implicated in both pulmonary and cardiovascular infections. Here, we report the draft genome sequences of three strains isolated from human respiratory specimens.

## GENOME ANNOUNCEMENT

*Mycobacterium chimaera* is a nontuberculous mycobacterium (NTM) of the *Mycobacterium avium* complex (MAC) associated with opportunistic infections in patients with underlying lung disease (1). More recently, *M. chimaera* has been identified as a causative agent of postoperative cardiovascular infections in several European countries (2, 3). In spite of its emerging clinical relevance, the genetics and pathophysiology of this species remain largely uncharacterized and whole-genome sequencing studies of this pathogen are yet to be described.

To gain insight into the genomics of this species, we undertook whole-genome sequencing of three clinical *M. chimaera* respiratory isolates, recovered from specimens at the Irish Mycobacteria Reference Laboratory (Dublin, Ireland). *M. chimaera* isolates were identified by sequence analysis of the 16S rRNA gene and 16S-23S rDNA spacer (ITS) region (4, 5). Resultant 16S and ITS sequences shared 100% identity with *M. chimaera* strain FI-0169T (AJ548480.2) originally described by Tortoli and colleagues (1). Total *M. chimaera* genomic DNA from each isolate was sequenced using a paired-end approach on an Illumina MiSeq instrument (TrinSeq, Trinity College, Dublin, Ireland). Sequence reads were quality-trimmed using Trimmomatic and assembled using Spades version 3.6.0 (6, 7). Resultant contigs were oriented to the *M. intracellulare* MOTT-02 genome (CP003323.1) using ABACAS (8). Gene annotation was performed using the NCBI Prokaryotic Genomes Automatic Annotation Pipeline (PGAAP; http://www.ncbi.nlm.nih.gov/genome/annotation_prok). The features and assembly statistics of all three *M. chimaera* isolates are detailed in Table 1.

**TABLE 1:** Genomic sequence assembly overview

Strain	Yr isolated	Specimen type	Total reads	Assembly size	Fold coverage	% G+C	Contigs (>2 kb)	*N*_50_ (bp)	Largest contig (bp)	No. of ORFs^a^	GenBank accession no.
MCIMRL2	2009	Sputum	3,466,168	6,087,047	50×	67.7	247	46,281	161,388	5,632	LJHL00000000
MCIMRL4	2013	Sputum	4,040,276	6,020,776	77×	67.7	210	89,969	201,826	5,553	LJHM00000000
MCIMRL6	2014	BAL^b^	3,334,536	6,451,412	67×	67.6	150	71,588	195,331	5,983	LJHN00000000

a ORFs, open reading frames.

b Bronchoalveolar lavage.

Comparative analysis of *M. chimaera* genome assemblies revealed an average nucleotide identity (ANI) of 99.9% between strains, consistent with their assignment to a common species (9). In contrast, a lower ANI of 97.7% was obtained when assemblies were compared to *M. intracellulare*—a closely related yet distinct species of the MAC. The observed ANI values correlate with observed divergence in the *M. chimaera* 16S rRNA gene and ITS regions relative to *M. intracellulare* and lend credence to the use of the 16S rRNA gene and ITS sequence analysis to distinguish *M. chimaera* clinically (1, 2, 5).

Analysis of reciprocal BLAST hits among non-pseudogene-coding sequences revealed a set of 4,951 genes common to all three *M. chimaera* isolates (10). Strains MCIMRL2 and MCIMRL6 exhibited a higher degree of similarity to each other than to MCIMRL4, sharing 5,230 genes, whereas MCIMRL4 shared 4,993 and 5,044 genes with MCIMRL2 and MCIMRL6, respectively. MCIMRL4 divergence was also reflected in comparative analysis of the 4,951 common gene sequences; MCIMRL4 diverged from MCIMRL2 and MCIMRL6 by 2,763 and 2,825 “core” single nucleotide variants (SNVs), respectively, whereas only 242 SNVs separated MCIMRL2 and MCIMRL6. Among the common genes shared by all three strains were putative host-interaction factors, including several conserved type-VII secretion systems and multiple PE/PPE/PE-GRS-family proteins, which represent important virulence determinants in other pathogenic mycobacteria (11).

This report represents the first whole-genome sequencing study of *M. chimaera*—an emerging opportunistic pathogen of the MAC. The data will serve as a useful reference for *M. chimaera* genomic epidemiology and provide the first insights into the potential virulence determinants of this pathogen.

### Nucleotide sequence accession numbers.

This whole-genome shotgun project has been deposited at DDBJ/EMBL/GenBank under the accession numbers listed in Table 1.

## References

[1] Tortoli E, Rindi L, Garcia MJ, Chiaradonna P, Dei R, Garzelli C, Kroppenstedt RM, Lari N, Mattei R, Mariottini A, Mazzarelli G, Murcia MI, Nanetti A, Piccoli P, Scarparo C 2004 Proposal to elevate the genetic variant MAC-A, included in the *Mycobacterium avium* complex, to species rank as *Mycobacterium chimaera* sp. nov. Int J Syst Evol Microbiol 54:1277–1285. doi:10.1099/ijs.0.02777-0. 15280303

[2] Sax H, Bloemberg G, Hasse B, Sommerstein R, Kohler P, Achermann Y, Rössle M, Falk V, Kuster SP, Böttger EC, Weber R 2015 Prolonged outbreak of *Mycobacterium chimaera* infection after open-chest heart surgery. Clin Infect Dis 61:67–75. doi:10.1093/cid/civ198. 25761866

[3] Kohler P, Kuster SP, Bloemberg G, Schulthess B, Frank M, Tanner FC, Rössle M, Böni C, Falk V, Wilhelm MJ, Sommerstein R, Achermann Y, Ten Oever J, Debast SB, Wolfhagen MJHM, Brandon Bravo Bruinsma GJ, Vos MC, Bogers A, Serr A, Beyersdorf F, Sax H, Böttger EC, Weber R, van Ingen J, Wagner D, Hasse B 2015 Healthcare-associated prosthetic heart valve, aortic vascular graft, and disseminated *Mycobacterium chimaera* infections subsequent to open heart surgery. Eur Heart J [Epub ahead of print.] doi:10.1093/eurheartj/ehv342. 26188001

[4] Roth A, Fischer M, Hamid ME, Michalke S, Ludwig W, Mauch H 1998 Differentiation of phylogenetically related slowly growing mycobacteria based on 16S-23S rRNA gene internal transcribed spacer sequences. J Clin Microbiol 36:139–147. 9431937 10.1128/jcm.36.1.139-147.1998 PMC124824

[5] Roth A, Reischl U, Streubel A, Naumann L, Kroppenstedt RM, Habicht M, Fischer M, Mauch H 2000 Novel diagnostic algorithm for identification of mycobacteria using genus-specific amplification of the 16S-23S rRNA gene spacer and restriction endonucleases. J Clin Microbiol 38:1094–1104. 10699003 10.1128/jcm.38.3.1094-1104.2000 PMC86348

[6] Bolger AM, Lohse M, Usadel B 2014 Trimmomatic: a flexible trimmer for Illumina sequence data. BioInformatics 30:2114–2120. doi:10.1093/bioinformatics/btu170. 24695404 PMC4103590

[7] Bankevich A, Nurk S, Antipov D, Gurevich AA, Dvorkin M, Kulikov AS, Lesin VM, Nikolenko SI, Pham S, Prjibelski AD, Pyshkin AV, Sirotkin AV, Vyahhi N, Tesler G, Alekseyev MA, Pevzner PA 2012 SPAdes: a new genome assembly algorithm and its applications to single-cell sequencing. J Comput Biol 19:455–477. doi:10.1089/cmb.2012.0021. 22506599 PMC3342519

[8] Assefa S, Keane TM, Otto TD, Newbold C, Berriman M 2009 ABACAS: algorithm-based automatic contiguation of assembled sequences. BioInformatics 25:1968–1969. doi:10.1093/bioinformatics/btp347. 19497936 PMC2712343

[9] Konstantinidis KT, Ramette A, Tiedje JM 2006 The bacterial species definition in the genomic era. Philos Trans R Soc Lond B Biol Sci 361:1929–1940. 17062412 10.1098/rstb.2006.1920 PMC1764935

[10] Altschul S, Madden TL, Schaffer AA, Zhang J, Zhang Z, Miller W, Lipman DJ 1997 Gapped BLAST and psi-blast: a new generation of protein database search programs. Nucleic Acids Res 25:3389–3402. doi:10.1093/nar/25.17.3389. 9254694 PMC146917

[11] Houben ENG, Korotkov KV, Bitter W 2014 Take five—type VII secretion systems of mycobacteria. Biochim Biophys Acta 1843:1707–1716. doi:10.1016/j.bbamcr.2013.11.003. 24263244

